# Pathophysiology of HFpEF: Insights from a Metabolic–Mitochondrial Perspective

**DOI:** 10.3390/ijms27010284

**Published:** 2025-12-26

**Authors:** Cristina Gatto, Maria Rosaria Rusciano, Valeria Visco, Carmine Vecchione, Michele Ciccarelli

**Affiliations:** 1 Department of Medicine, Surgery and Dentistry, University of Salerno “Scuola Medica Salernitana”, 84081 Baronissi, Italy; crgatto@unisa.it (C.G.); mrusciano@unisa.it (M.R.R.); vvisco@unisa.it (V.V.); cvecchione@unisa.it (C.V.); 2 Scuola di Specializzazione in Patologia Clinica e Biochimica Clinica, University of Salerno “Scuola Medica Salernitana”, 84081 Baronissi, Italy

**Keywords:** HFpEF, metabolic flexibility, mitochondria, oxidative stress, inflammation

## Abstract

Heart failure with preserved ejection fraction (HFpEF) represents a growing clinical challenge, accounting for more than half of all cases of heart failure, for which there are currently no effective treatments. Emerging evidence identifies mitochondrial dysfunction as a central mechanism linking metabolic comorbidities, systemic inflammation, and energy failure in HFpEF. This review provides a comprehensive overview of the metabolic–mitochondrial mechanisms underlying the pathophysiology of HFpEF. Loss of metabolic flexibility, characterized by reduced fatty acid and glucose oxidation, leads to energy inefficiency, lipid accumulation, and oxidative stress. Structural and functional mitochondrial abnormalities, including damaged cristae, altered fission-fusion dynamics, and impaired oxidative phosphorylation, contribute to diastolic dysfunction and ventricular remodeling. In parallel, chronic inflammation and redox imbalance amplify mitochondrial damage through cytokine- and ROS-mediated pathways, creating a cycle of bioenergetic failure. From a therapeutic perspective, strategies aimed at restoring mitochondrial homeostasis, such as physical training, metabolic modulation, SGLT2 inhibition, ketone supplementation, and mitochondria-targeted antioxidants, show promising preclinical results. However, clinical translation remains limited. Deepening the understanding of mitochondrial metabolism could enable the development of personalized treatments capable of improving outcomes for HFpEF patients.

## 1. Introduction

Heart failure (HF) is a complex clinical syndrome characterized by structural and/or functional abnormalities of the myocardium, ultimately impairing the ventricle’s ability to fill or pump blood efficiently. HF is categorized into four phenotypes based on left ventricular ejection fraction (LVEF) according to the recent guidelines: LVEF ≤ 40% for HFrEF (Reduced EF), LVEF 41–49% for HFmrEF (Mildly Reduced EF), LVEF ≥ 50% for HFpEF (Preserved EF), and HFimpEF (Improved EF) [1,2]. Although they share common clinical manifestations, such as dyspnea, fatigue, and exercise intolerance, their underlying pathophysiology, patient characteristics, and therapeutic responses are markedly different.

HFrEF is primarily caused by loss of contractile function following myocardial injury, including ischemic heart disease or dilated cardiomyopathy, resulting in systolic dysfunction and ventricular dilation. In contrast, HFpEF is characterized by preserved systolic performance but impaired diastolic relaxation, increased myocardial stiffness, and elevated filling pressures. HFpEF is typically associated with aging, obesity, hypertension, and type 2 diabetes mellitus, conditions that collectively create a systemic proinflammatory and metabolic state [3].

HFpEF has become one of the most challenging conditions in cardiology due to its increasing prevalence, complex causes, and limited treatment options [4]. The syndrome is characterized by a normal or nearly normal LVEF, combined with impaired diastolic relaxation, higher filling pressures, and increased ventricular stiffness [5,6]. According to the Echocardiography and Heart Failure Associations of the European Society of Cardiology, diagnostic criteria for HFpEF include: (i) clinical signs or symptoms of HF; (ii) evidence of preserved systolic function; and (iii) objective proof of diastolic dysfunction [7].

Clinically, patients frequently present with exercise intolerance, pulmonary congestion, and signs of systemic volume overload despite maintained ejection fraction. Epidemiological data indicate that HFpEF now accounts for over half of all HF cases, a trend driven by population aging and the global rise in metabolic and cardiovascular comorbidities [8,9]. Notably, while multiple evidence-based therapies, such as neurohormonal antagonists and sodium–glucose cotransporter 2 (SGLT2) inhibitors, have significantly improved outcomes in HFrEF, no treatment has efficacy in reducing mortality among HFpEF patients. This highlights the need for a deeper mechanistic understanding and phenotype-specific therapeutic approaches.

In recent years, the conceptual framework of HFpEF has shifted from viewing it as a disease limited to the heart to identifying it as a multi-system disorder. This change reflects complex interactions among the cardiovascular, metabolic, and immune systems [10,11]. Low-grade chronic inflammation, endothelial dysfunction, and oxidative stress are recognized as key mechanisms connecting systemic metabolic abnormalities to cardiac remodeling, fibrosis, and diastolic dysfunction [12,13,14]. Mitochondria, essential organelles responsible for energy production, redox regulation, and cell survival, have become central players in the development and progression of HFpEF in this pathophysiological context.

Mitochondrial dysfunction in HFpEF involves multiple mechanisms, including impaired oxidative phosphorylation, altered substrate utilization, reduced ATP production, and excessive production of reactive oxygen species (ROS) [15,16,17]. These alterations not only compromise cardiomyocyte bioenergetics but also activate maladaptive signaling pathways that promote fibrosis, hypertrophy, and adverse ventricular remodeling [18].

Moreover, mitochondrial dysfunction is not limited to cardiomyocytes. Recent evidence indicates that endothelial cells, fibroblasts, and infiltrating immune cells also exhibit mitochondrial dysfunction in HFpEF, contributing to systemic inflammation, endothelial activation, and metabolic stress [19]. Impaired mitochondrial quality control mechanisms, such as defective fission–fusion dynamics and dysregulated mitophagy, further exacerbate energetic dysregulation, oxidative damage, and cellular senescence [16,20]. In endothelial cells, mitochondrial dysfunction leads to reduced nitric oxide bioavailability, microvascular alterations that contribute to myocardial stiffness, and impaired diastolic function [21].

Understanding the molecular underpinnings of mitochondrial dysfunction in HFpEF is therefore essential to identify novel therapeutic targets and develop mechanism-based interventions. This review aims to summarize current evidence on the role of mitochondrial dysfunction in HFpEF; explore the mechanistic links between mitochondrial, metabolic, and inflammatory pathways; and discuss emerging therapeutic strategies aimed at restoring mitochondrial homeostasis and improving cardiac performance.

## 2. Loss of Metabolic Flexibility

The adult heart is a highly energy-demanding organ that primarily relies on mitochondrial oxidative phosphorylation to maintain its continuous contractile activity [22]. Under normal physiological conditions, about 60–90% of myocardial ATP is produced through the oxidation of fatty acids, with the remaining portion coming from the oxidation of glucose, lactate, amino acids, and ketone bodies [15,23]. This metabolic setup provides the heart with metabolic flexibility, enabling it to adjust its substrate use based on the availability of substrates, hormonal signals, and energy needs, thereby preserving contractile efficiency and blood circulation [24].

**Figure 1:**
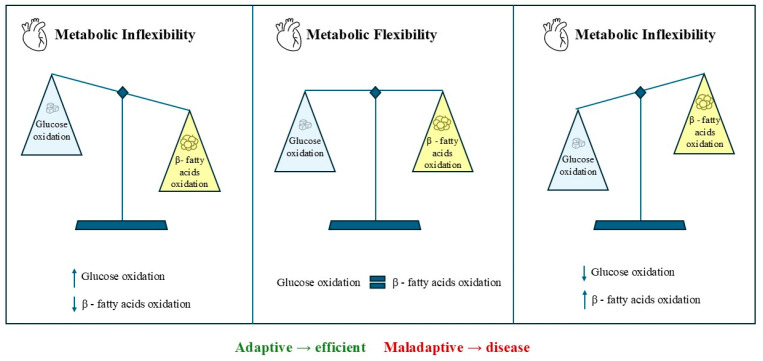
Metabolic Flexibility in the Heart.

In the context of HF, however, this flexibility gradually decreases. Failing hearts often develop insulin resistance and a metabolic shift toward greater reliance on anaerobic glycolysis, rather than oxidative phosphorylation, leading to lower energy efficiency and increased lactate storage [25]. As a result, the myocardium in HFpEF experiences harmful metabolic reprogramming characterized by a shift from efficient fatty acid oxidation to higher glucose utilization. Although initially a compensatory response, this metabolic change is associated with decreased mitochondrial oxidative capacity, impaired ATP production, and overall bioenergetic inefficiency (Figure 1).

Over time, this metabolic stress causes toxic lipid intermediate accumulation, mitochondrial dysfunction, and oxidative damage, which further worsen contractile function and diastolic performance [26,27]. Patients with HFpEF, indeed, have significantly more intramyocardial lipids than HFrEF patients or non-HF controls. A study using magnetic resonance imaging on HFpEF, HFrEF, and healthy subjects showed higher cardiac lipid content in HFpEF individuals [28]. Conversely, numerous animal studies have shown a relationship between cardiac steatosis and cardiac remodeling. Lipid accumulation leads to the formation of toxic intermediates and apoptosis [29]. This loss of metabolic flexibility is now recognized as a key feature of energetic failure in HFpEF and as a potential target for therapies aimed at restoring mitochondrial function and enhancing cardiac performance.

Recent experimental studies have provided mechanistic insights into the metabolic changes underlying the pathophysiology of HFpEF. In HFpEF, the myocardium may exhibit two distinct metabolic profiles: one characterized by enhanced fatty acid uptake and oxidation, coupled with reduced glucose utilization, and another marked by diminished fatty acid oxidation, along with impaired glucose oxidation [30]. It therefore appears that alterations in fatty acid metabolism in patients with heart failure depend on the associated pathological context. In cases of HF linked to metabolic comorbidities, an increase in fatty acid oxidation is generally observed. Conversely, in HF associated with hypertension or ischemia, fatty acid oxidation tends to decrease [23].

In a murine “two-hit” model of HFpEF, combining a high-fat diet with nitric oxide synthase inhibition, the myocardium shows a significant reprogramming of energy metabolism. Specifically, insulin-stimulated glucose oxidation was greatly reduced, while fatty acid oxidation was significantly increased, with no changes in ketone oxidation or glycolytic flux. Myocardial ATP production became mainly dependent on fatty acid oxidation, highlighting a loss of the normal balance between substrate utilization pathways [31]. Similar findings were reported in another “two-hit” HFpEF murine model, where isolated cardiac mitochondria showed reduced respiration rates when fueled with palmitoyl carnitine or pyruvate, indicating alterations in fatty acid β-oxidation and pyruvate oxidative metabolism. These impairments suggest that mitochondrial dysfunction plays a key role in the bioenergetic inefficiency seen in HFpEF, linking altered substrate oxidation to decreased ATP generation and contractile dysfunction [32].

A study involving 168 patients examined how the acylcarnitine-to-free carnitine (AC/FC) ratio influences heart failure outcomes. Carnitine is essential for transporting fatty acids into mitochondria for beta-oxidation. Among these patients, a higher AC/FC ratio was associated with a greater risk of adverse cardiac events. This association was significant in patients with HFpEF but not in those with HFrEF, indicating that fatty acid oxidation is impaired in HFpEF [33]. It should be noted that studies on the effect of angiotensin II (Ang II) in HFpEF models have shown a reduction in glucose oxidation through the upregulation of pyruvate dehydrogenase kinase 4, while fatty acid oxidation remains unchanged or slightly decreased, rather than increasing to compensate for reduced glucose utilization. This suggests that metabolic flexibility in HFpEF is a rather complex process regulated by several factors [34].

Taken together, these findings emphasize that metabolic inflexibility and reduced substrate oxidation are central features of energy deficiency in HFpEF. The resulting imbalance in substrate utilization not only limits ATP availability but also increases oxidative stress and lipid accumulation, creating conditions for mitochondrial damage. These metabolic imbalances thus provide a mechanistic link between impaired energy metabolism and the structural and functional mitochondrial abnormalities that characterize HFpEF.

## 3. Mitochondrial Structural and Functional Alterations

The structural integrity of mitochondria is essential for maintaining efficient energy production in cardiomyocytes. To preserve cellular homeostasis, cells possess highly regulated mechanisms that ensure the removal of dysfunctional mitochondria and the renewal of healthy ones. This dynamic quality control occurs through the opposing processes of mitochondrial fission and fusion, which together maintain the morphology, distribution, and functionality of the organelle. A fine balance between these two processes is essential; disruption of this balance leads to mitochondrial dysfunction, impaired oxidative phosphorylation, and ultimately, compromised cellular energy [35].

In HFpEF, alterations in mitochondrial ultrastructure have been consistently observed. However, whether mitochondrial dysfunction is the cause or effect of the structural alterations found in HFpEF remains to be clarified [17].

Electron microscopy of human HFpEF myocardial samples showed ultrastructural abnormalities, including mitochondrial swelling, disrupted cristae, and lipid droplet buildup next to mitochondria, indicating lipotoxic stress and impaired β-oxidation [36]. Additionally, changes in mitochondrial fission and fusion machinery, specifically increased dynamin-related protein 1 (DRP1)-driven fission and decreased mitofusin 2 (MFN2)-driven fusion, led to mitochondrial fragmentation and cardiomyocyte apoptosis [37]. Consistently, a study by Givvimani et al., demonstrates that DRP-1 inhibition is associated with an improvement in LV dysfunction and decreased levels of LC-3 and p62 [38].

Proteomic and metabolomic analyses have also shown downregulation of key enzymes involved in the tricarboxylic acid cycle and electron transport chain, including reduced activity of complexes I and IV. These changes result in decreased oxidative phosphorylation and increased ROS production, thereby promoting oxidative stress and triggering maladaptive signaling that drives fibrosis and hypertrophy [39].

Mitochondria play a central role in regulating acute diastolic function through two closely interconnected mechanisms: mitochondrial calcium management and bioenergetic support for calcium reuptake. During systole, mitochondria uptake Ca^2+^ via the mitochondrial calcium uniporter (MCU), a process that contributes to transient cytosolic Ca^2+^ buffering while simultaneously stimulating oxidative phosphorylation. Under conditions of ischemia or acute metabolic stress, mitochondrial Ca^2+^ uptake is impaired, resulting in cytosolic Ca^2+^ accumulation and delayed cardiomyocyte relaxation. At the same time, efficient diastolic relaxation critically depends on adequate ATP availability to support Ca^2+^ reuptake into the sarcoplasmic reticulum via SERCA2a, a process that is highly dependent on ATP. Acute mitochondrial dysfunction, such as occurring during myocardial ischemia, leads to reduced ATP production, directly compromising SERCA2a activity. This results in incomplete clearance of Ca^2+^ from the sarcomere during diastole, thus causing acute diastolic dysfunction [40]. A study on the mouse model of HFpEF showed that cardiac mitochondria exhibit swelling and a significant decrease in Ca^2+^ content, linked to a reduction in mitochondrial respiration. This reduction leads to a decrease in SERCA2a and NCX activity, which in turn causes atrial arrhythmias, one of the most common complications in HFpEF patients [41].

Chaanine et al., investigated mitochondrial morphology, dynamics, and function, demonstrating that, in terms of ultrastructure, HFpEF patients are characterized by mitochondrial fragmentation, cristae destruction, vacuolar degeneration, and decreased mitochondrial area [42]. These morphological changes could be explained by the BNIP3 levels. BNIP3 is a natural inhibitor of Optic Atrophy 1 (OPA1), involved in the fusion of the inner mitochondrial membrane and in maintaining the structure of mitochondrial cristae [43]. Similarly, Bode et al. observed increased mitochondrial fission in left atrial (LA) cardiomyocytes of obese ZFS-1 rats in a model of metabolic HFpEF [44]. Similar results were reported in a hypertensive HFpEF model (salt-sensitive Dahl rat), where multi-omic analysis showed that inflammatory response and mitochondrial fission are the main processes involved in myocyte stiffening [45].

These abnormalities are found not only in the heart but also in related organs connected to the cardiovascular system. In skeletal muscles of HFpEF patients, similar mitochondrial derangements are observed, characterized by reduced oxidative capacity and lower mitochondrial density, contributing to exercise intolerance and systemic metabolic inefficiency [19]. Furthermore, Molina et al. highlighted that in elderly patients with HFpEF, muscle tissue has reduced mitochondrial content and low levels of MFN2, alterations closely related to reduced aerobic capacity, measured as maximum VO2, compared to control subjects [46]. However, as pointed out by Martinez et al., it should be investigated whether the alterations in the fission process are due to altered oxygen consumption by the muscles or whether they are a direct consequence of the mitochondrial dysfunction underlying HFpEF [47].

Overall, evidence indicates that structural and functional alterations in mitochondria in HFpEF underline a widespread energy deficit involving both the heart and other metabolically active tissues, highlighting the importance of strategies aimed at improving mitochondrial bioenergetics.

## 4. Inflammation, Oxidative Stress, and Mitochondrial Damage

**Figure 2:**
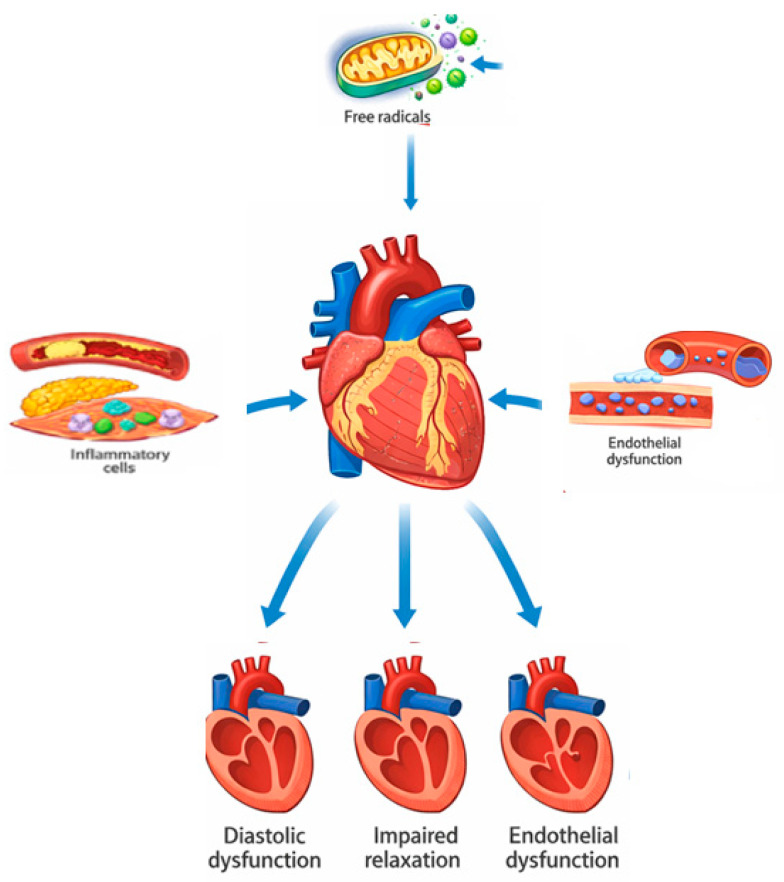
Mechanistic aspect of HFpEF.

HFpEF is increasingly recognized as an inflammatory-metabolic disease, in which systemic comorbidities trigger endothelial inflammation and oxidative stress that compromise myocardial function [11,12]. Conditions such as obesity, diabetes mellitus, metabolic syndrome, and hypertension create a systemic inflammatory environment characterized by elevated levels of circulating cytokines, adipokine imbalance, and endothelial activation (Figure 2).

These systemic disturbances lead to coronary microvascular inflammation, reduced nitric oxide (NO) bioavailability, and impaired myocardial relaxation, contributing to the pathophysiology of HFpEF. The contribution of inflammation has been convincingly demonstrated in a porcine model, in which the coexistence of hypertension, diabetes mellitus, and hypercholesterolemia induces diastolic dysfunction and heart failure even in the absence of coronary artery disease [48]. This reinforces the concept that inflammation caused by comorbidity, rather than coronary ischemia alone, plays a central role in the onset of HFpEF.

Proinflammatory cytokines such as IL-6, TNF-α, and CRP are consistently elevated in patients with HFpEF and are correlated with disease severity, diastolic dysfunction, and exercise intolerance [13]. These cytokines exert multiple harmful effects on the myocardium: they activate cardiac fibroblasts, stimulate extracellular matrix (ECM) deposition, and promote the development of diffuse interstitial fibrosis, leading to increased ventricular stiffness. In addition, systemic inflammatory markers such as the neutrophil/lymphocyte ratio, which reflects the balance between innate and adaptive immune activation, are also associated with worse clinical outcomes in HFpEF [49]. Chronic diastolic dysfunction is dominated by structural remodeling rather than impaired calcium kinetics alone. Persistent injury, pressure overload, or metabolic disease leads to incomplete myocardial repair, in which damaged cardiomyocytes are replaced by fibrosis rather than functional muscle. This process is strongly influenced by the immune system, particularly macrophage phenotype, chronic low-grade inflammation, and dysregulated fibroblast activation. Aging further amplifies these mechanisms, as senescence favors collagen deposition, reduced matrix turnover, and increased myocardial stiffness. The result is a noncompliant ventricle with preserved or mildly reduced systolic function but elevated filling pressures [50].

During early cardiac stress, including pressure or volume overload, there is a marked increase in endothelial activation, with increased expression of adhesion molecules (e.g., VCAM-1, ICAM-1) and increased production of chemokines and cytokines within the myocardial microenvironment [51]. This pro-inflammatory state facilitates the recruitment and infiltration of inflammatory cells, particularly monocytes and macrophages. Such myocardial infiltration, well documented in models of hypertension and HFpEF [52]. contributes to microvascular dysfunction, impaired myocardial perfusion, and progressive fibrosis. Human ventricular biopsies from hypertensive and HFpEF patients confirm a significantly higher macrophage density compared to healthy controls [53]. The presence of NOX2-producing macrophages and high CD68 expression further support the role of innate immune activation in HFpEF, as demonstrated in both human samples and the ZSF1-HFpEF animal model [52].

Inflammation directly affects mitochondrial function, creating a bidirectional vicious cycle. Mitochondria are both the main producers and targets of ROS, and inflammatory cytokines such as TNF-α and IL-1β compromise several components of the electron transport chain (ETC), increasing ROS generation and promoting oxidative damage to lipids, proteins and mitochondrial DNA (mtDNA) [15]. Endothelial cells are similarly affected: cytokine-mediated activation of NADPH oxidase leads to excessive superoxide production, which rapidly scavenges NO and reduces its bioavailability [54]. Elevated ROS levels also induce eNOS uncoupling, shifting its enzymatic activity from NO production to superoxide (O_2_^−^) generation, thereby exacerbating vascular dysfunction and reducing vasodilatory capacity [55]. This redox imbalance represents a central mechanism underlying the chronically impaired endothelial function observed in HFpEF.

Oxidative stress occurs when there is an increase in ROS production and a decrease in the antioxidant defense system [56]. This imbalance is a well-established contributor to the onset of various cardiovascular diseases and maladaptive cardiac remodeling observed in HFpEF patients [57].

Patients with HFpEF show significant bioenergetic dysfunction characterized by reduced oxidative phosphorylation and impaired mitochondrial coupling. This change leads to increased electron leakage, causing an overload of reactive species [26]. Additionally, in obese experimental models, mitochondrial respiration driven by complex I is especially impaired. Since complex I is the main source of mitochondrial ROS, its downregulation is strongly linked to increased oxidative stress and triggers apoptosis signaling via the JNK pathway [58]. Mitochondrial ROS boosts inflammatory pathways by activating NF-κB and NLRP3 inflammasome, creating a cycle of ongoing inflammation and oxidative damage that worsens myocardial injury and promotes HFpEF-related remodeling [20]. The NLRP3 inflammasome is now widely recognized as a key molecular hub connecting mitochondrial dysfunction with innate immune activation. Notably, its assembly and activation are closely regulated by the cellular redox state, as oxidative conditions directly influence NLRP3’s dynamics and downstream signaling [59].

These findings emphasize that mitochondrial dysfunction serves as a key factor in promoting both oxidative stress and inflammation. These interconnected processes work together to drive the development of diastolic dysfunction and the cardiac remodeling features of HFpEF.

## 5. Therapeutic and Translational Implications

The main therapeutic goals in managing HFpEF are to relieve symptoms, enhance quality of life, prevent disease progression, and decrease hospitalization rates, while also addressing associated comorbidities.

In this context, lifestyle changes and physical activity are essential parts of overall treatment strategies. Numerous studies have demonstrated that physical training [60], exercise-based cardiac rehabilitation, and weight loss improve cardiorespiratory function, functional capacity, and quality of life, while reducing hospital admissions [61]. Additionally, dietary changes, such as sodium restriction, have been shown to improve ventricular diastolic function and arterial elasticity in hypertensive patients with HFpEF [62].

The pharmacological therapy is based on diuretics, angiotensin receptor–neprilysin inhibitors (ARNIs), sacubitril/valsartan, angiotensin receptor blockers (ARBs), and mineralocorticoid antagonists (MRAs), especially in patients with elevated blood pressure levels [63]. The PARADIGM-HF research determined whether sacubitril-valsartan, the first medication of the angiotensin II receptor neprilysin inhibitor (ARNI) class, was more effective than enalapril in improving survival and lowering hospitalization rates in a subset of heart failure patients with reduced ventricular function [64]. PIONEER-HF Comparison of Sacubitril/Valsartan Versus Enalapril on Effect on N-terminal pro-B-type natriuretic peptide in Patients Stabilized from an Acute HF Episode was a multicenter, randomized, double-blind trial of in-hospital initiation of sacubitril/valsartan versus enalapril. The results of this study demonstrate that the risk of cardiovascular death or rehospitalization for HF in these subgroups is modifiable with in-hospital initiation of sacubitril/valsartan [2].

More recently, SGLT-2, initially used for diabetes management, has emerged as a drug that can reduce the hospitalization rate. In a study published by Solomon et al., dapagliflozin reduced the risk of worsening heart failure or cardiovascular death among patients with HFpEF, improving patients’ outcomes [65]. Consistently, in the EMPEROR study, 5988 patients with class II-IV heart failure and an ejection fraction greater than 40% received empagliflozin or placebo in addition to their usual therapy. The study demonstrated that empagliflozin reduced the combined risk of cardiovascular death or hospitalization for heart failure in patients with heart failure and preserved ejection fraction, regardless of whether they had diabetes or not [66].

In addition to traditional neurohormonal blockades, ranolazine acts as a pharmacological agent that directly targets diastolic dysfunction and myocardial energy imbalance. By selectively inhibiting late inward sodium currents, ranolazine reduces pathological intracellular sodium accumulation, thereby limiting secondary calcium overload. This mechanism results in improved active relaxation, reduced diastolic wall tension, and increased ventricular compliance, regardless of heart rate reduction. Ranolazine exerts favorable metabolic effects, including modest but consistent reductions in glycated hemoglobin (HbA1c), suggesting improved myocardial and systemic glucose utilization. This metabolic modulation may be particularly relevant in cardiometabolic phenotypes characterized by insulin resistance, microvascular dysfunction, and low-grade inflammation, where energy inefficiency contributes to maladaptive remodeling. In contrast to β-blockers, which may prolong diastolic filling time and peripheral tolerance, ranolazine improves diastolic performance without negatively impacting blood pressure. Its clinical benefits appear especially pronounced in conditions dominated by impaired relaxation, microvascular ischemia, and interstitial fibrosis, supporting the concept that late sodium current inhibition represents a mechanistically distinct and complementary strategy within contemporary heart failure therapeutics [67].

Despite these properties, ranolazine remains largely confined to the treatment of chronic angina, and its potential role in HFpEF, diabetic cardiomyopathy, and fibrotic remodeling has been insufficiently explored. A renewed interest in ranolazine, particularly in combination with agents targeting fibrosis, lymphatic function, and neurohormonal activation, may open novel therapeutic avenues for patients in whom diastolic dysfunction represents the dominant pathophysiological substrate.

Beyond the classical pharmacological interventions, therapeutic strategies aimed at improving mitochondrial health have attracted considerable interest. Exercise training remains the most effective non-pharmacological intervention. A study on animal models showed that 8 weeks of physical exercise after myocardial infarction was able to restore the number and size of mitochondria and enhance oxidative capacity [68]. Moreover, exercise has been shown to restore the balance between fusion and fission by restoring MFN levels, generally altered in HFpEF patients, thereby improving overall mitochondrial function [41]. Experimental evidence demonstrates that in the rat heart, exercise training increases antioxidant enzyme activity while simultaneously reducing the ROS production [69].

HFpEF is characterized by a metabolic shift and an imbalance in substrate utilization, which contributes to impaired myocardial energy [70]. Consequently, strategies aimed at modulating cardiac metabolism have emerged as a promising therapeutic approach to restore energy homeostasis and improve cardiac function.

Ketone bodies have emerged as an efficient alternative substrate for the failing heart. Ketone oxidation enhances cardiac energetics and reduces ROS production; in animal models of HFpEF, β-hydroxybutyrate supplementation improved diastolic relaxation and mitochondrial respiration [71]. In patients with type 2 diabetes (T2D) and HFpEF, a two-week treatment with oral ketone supplementation was shown to enhance cardiac output while reducing cardiac filling pressures and ventricular stiffness, suggesting an improvement in diastolic function and overall cardiac efficiency [72].

Among pharmacology strategies, SGLT2 inhibitors, in addition to improving the phenotype of HFpEF patients, have important effects at the mitochondrial level. Mechanistically, they promote mild ketonemia, weight loss, and improved mitochondrial redox state. In the failing heart, SGLT2i promote a reduction in inflammatory processes through the inhibition of the nucleotide-binding domain-like receptor protein 3 (NLRP3) inflammasome and inflammatory biomarkers, including IL-6 and C-reactive protein [73,74]. From an energy perspective, it has been hypothesized that SGLT2 inhibitors promote an increase in ketone body production, thereby improving cardiac mitochondrial function and reducing oxidative stress, which results in enhanced overall myocardial performance [75]. In preclinical HFpEF models, empagliflozin enhances autophagy via the AMPK/mTORC1 pathway [76]. Notably, in an obese rat model of HFpEF, dual SGLT-1 and SGLT-2 inhibition prevents mitochondrial swelling, enhances mitochondrial Ca^2+^ buffer capacity, improves mitochondrial fission, and ROS production [77].

Mitochondrial dysfunction contributes to both myocardial and vascular diastolic impairment through alterations in ATP production, redox homeostasis, and calcium management. Consequently, mitochondria have emerged as an interesting therapeutic target in conditions characterized by diastolic dysfunction. Among emerging approaches, photobiomodulation (PBM), i.e., the application of specific wavelengths of light, predominantly in the red and near-infrared range, has been shown in experimental models to modulate mitochondrial function. Cytochrome c oxidase, a terminal enzyme in the mitochondrial electron transport chain, has been identified as the main photoacceptor, with light exposure influencing mitochondrial membrane potential, ATP generation, and reactive oxygen species-dependent signaling pathways [78,79].

Additional promising compounds include mitochondria-targeted antioxidants designed to stabilize cardiolipin, preserve the integrity of the electron transport chain (ETC) and reduce excessive mitochondrial ROS production, three processes that are profoundly altered in HFpEF. By selectively accumulating within mitochondria, these molecules aim to counteract oxidative damage, thereby preventing downstream effects such as mtDNA oxidation, impaired ATP synthesis, and activation of pro-inflammatory signaling pathways. AMPK activators represent another therapeutic avenue, as AMPK serves as a key regulator of cellular energy balance. Enhancing AMPK activity restores metabolic flexibility, promotes fatty acid oxidation, stimulates mitochondrial biogenesis, and improves endothelial function, mechanisms central to the metabolic-inflammatory axis characteristic of HFpEF phenotypes [15].

Among the antioxidants targeting mitochondria, MitoQ is one of the most studied. MitoQ is a modified form of ubiquinone conjugated with a lipophilic triphenylphosphonium cation, which allows it to accumulate within the mitochondrial matrix. This enhances its antioxidant and anti-apoptotic effects by enabling efficient elimination of ROS in the inner mitochondrial membrane, which is particularly rich in cardiolipin, also essential for the assembly of ETC supercomplexes [80]. Preclinical studies have shown that pharmacological elimination of ROS with MitoQ preserves mitochondrial membrane potential, reduces lipid peroxidation, attenuates fibrosis and improves cardiac function in several animal models of heart failure. In particular, the cardioprotective benefits appear to extend beyond mitochondrial redox regulation, suggesting broader improvements in mitochondrial metabolism control and microvascular homeostasis [81]. Moreover, clinical data indicate that dietary supplementation with MitoQ in healthy elderly individuals improved endothelial function, suggesting potential vascular benefits beyond heart failure [82].

Translational research also supports the therapeutic potential of NAD^+^ precursors, such as nicotinamide riboside (NR) and nicotinamide mononucleotide (NMN). Declining NAD^+^ levels are a hallmark of aging, metabolic disease, and mitochondrial dysfunction, all of which contribute to HFpEF. NAD^+^ acts as a cofactor for key metabolic enzymes and sirtuins involved in oxidative phosphorylation, mitochondrial biogenesis, and antioxidant defense. Restoring NAD^+^ availability may therefore lead the myocardium towards a more energy-efficient and anti-inflammatory state [83]. In a mouse model of HFpEF, Tong et al. demonstrated that supplementation with NR or pharmacological activation of NAD^+^ biosynthesis improved mitochondrial respiration, reduced myocardial ROS, improved diastolic relaxation, and ultimately reversed several features of the HFpEF phenotype [32]. These studies provide compelling evidence that mitochondrial NAD^+^ metabolism can improve the energy deficit and oxidative stress that characterize HFpEF, particularly in metabolic and age-related subtypes.

Despite promising results observed in preclinical studies, translating these therapeutic approaches into clinical practice is difficult. This is largely due to the great heterogeneity of patients with HFpEF, who have different comorbidities, etiologies, and pathophysiological mechanisms, as well as the multifactorial nature of the syndrome, which involves complex interactions.

## 6. Challenges and Future Directions

The complexity of HFpEF stems from its marked heterogeneity. Patients may present distinct phenotypes determined by differences in age, sex, comorbidities such as obesity, diabetes, hypertension, chronic kidney disease, or atrial fibrillation, as well as divergent molecular and metabolic characteristics (Table 1). Current diagnostic algorithms focus primarily on structural and functional cardiac changes (e.g., left ventricular hypertrophy, diastolic dysfunction, left atrial enlargement), but do not adequately detect the metabolic, inflammatory, and mitochondrial abnormalities that underlie many subtypes of HFpEF. This gap leads to diagnostic uncertainty and limits the accuracy of treatment decisions.

The integration of mitochondrial biomarkers into clinical workflows can significantly improve phenotyping. Circulating mitochondrial DNA (mtDNA), for example, reflects mitochondrial damage and systemic inflammation, while acylcarnitine profiles provide information on altered fatty acid oxidation and overall metabolic inflexibility. Additional markers, such as cardiolipin, mitochondrial-derived peptides, and plasma oxidative stress indices, could complement cardiac imaging and natriuretic peptide levels, offering a more comprehensive characterization of disease mechanisms. Ultimately, an approach enriched by these biomarkers could help identify patients with predominant mitochondrial dysfunction who may benefit from targeted metabolic therapies.

A major obstacle to mechanistic understanding remains the limited availability of human myocardial tissue from patients with HFpEF, particularly in its different phenotypes. To overcome this obstacle, future research should increasingly rely on induced pluripotent stem cell-derived cardiomyocytes (iPSC-CMs), engineered cardiac tissues, and organoid models. These platforms allow for controlled analysis of mitochondrial respiration, reactive oxygen species generation, calcium handling, and metabolic reorganization. When combined with multi-omics strategies, including genomics, epigenomics, transcriptomics, proteomics, and metabolomics, they can reveal mitochondrial signaling pathways and regulatory networks specific to each HFpEF subtype.

Furthermore, large-scale, phenotype-stratified clinical trials are urgently needed to determine whether targeting mitochondrial bioenergetics, through agents that enhance oxidative phosphorylation, potentiate mitochondrial biogenesis, or reduce mitochondrial oxidative stress, can translate into meaningful clinical benefits. Such studies may require biomarkers to effectively capture therapeutic responses in distinct mitochondrial subgroups.

Finally, the development of non-invasive imaging methods capable of assessing mitochondrial function in vivo is a long-term goal. Techniques such as hyperpolarized magnetic resonance spectroscopy, PET tracers for mitochondrial membrane potential or oxidative metabolism, and advanced metabolic magnetic resonance imaging could enable real-time monitoring of mitochondrial activity within the myocardium. If successfully implemented, these tools would revolutionize both diagnosis and therapeutic surveillance, allowing clinicians to directly assess the impact of mitochondrial-targeted therapies in patients with HFpEF.

## 7. Conclusions

Mitochondrial dysfunction is a key and unifying mechanism that connects systemic metabolic comorbidities, chronic inflammation, and myocardial energy deficiency in HFpEF. In patients with obesity, insulin resistance, type 2 diabetes, or hypertension, systemic metabolic imbalances cause increased circulating lipids, endothelial dysfunction, and low-grade inflammation. These systemic abnormalities significantly impact cardiomyocyte mitochondria, impairing metabolic flexibility and creating an environment susceptible to oxidative stress. As a result, mitochondrial dysfunction not only indicates the presence of comorbidities but also actively contributes to myocardial stiffness, microvascular inflammation, and diastolic dysfunction—hallmarks of HFpEF.

A growing body of evidence from clinical studies, myocardial biopsies, animal models, and in vitro research highlights several specific mitochondrial defects in HFpEF:

(1) Altered substrate utilization with a shift from fatty acid oxidation to less efficient glucose metabolism, reflecting reduced metabolic adaptability;
(2) Impaired oxidative phosphorylation (OXPHOS) and reduced activity of electron transport chain complexes, contributing to inadequate ATP generation and impaired cardiomyocyte relaxation;
(3) Disturbed mitochondrial dynamics, including unbalanced fusion–fission processes and impaired mitophagy, which hinder mitochondrial turnover and quality control;
(4) Redox imbalance, characterized by excessive ROS production and antioxidant system inefficiency, which ultimately exacerbates cardiomyocyte dysfunction through oxidative damage to proteins, lipids, and mtDNA.

Despite recognition of the involvement of mitochondrial metabolism, current therapies for HFpEF remain limited to symptom control and risk factor management, with minimal effects on disease progression. Targeting mitochondrial health offers a promising therapeutic avenue. Lifestyle interventions, such as structured aerobic exercise and calorie restriction, have been shown to improve mitochondrial biogenesis, increase metabolic flexibility, and restore redox balance. Pharmacological strategies are also emerging, including modulators of mitochondrial metabolism (e.g., ketone therapies, AMPK activators), agents that promote mitochondrial quality control (e.g., mitophagy enhancers), and compounds that reduce oxidative stress.

However, translating these approaches into routine clinical practice requires a deeper understanding of how mitochondrial dysfunction varies among HFpEF phenotypes. Personalized therapy will depend on the ability to identify patients whose disease is primarily caused by mitochondrial abnormalities. This highlights the need for validated non-invasive mitochondrial biomarkers, such as circulating mtDNA, acylcarnitine signatures, peptides of mitochondrial origin, and advanced metabolic imaging tools capable of assessing mitochondrial function in vivo.

Future research should prioritize improving mitochondrial bioenergetics, whether through lifestyle, pharmacological or device-based interventions, in order to achieve clinically meaningful outcomes, including improved quality of life, reduced hospitalization rates and increased long-term survival. Bridging the gap between preclinical knowledge and patient-centered outcomes is essential for identifying mitochondria as both a diagnostic tool and a therapeutic target in HFpEF.

## Figures and Tables

**Table 1:** Summary of key mitochondrial mechanisms contributing to the pathophysiology of HFpEF and therapeutic strategies.

HFpEF Alterations	Pathophysiological Features	Experimental/Clinical Evidence
Loss of Metabolic Flexibility	↓ Fatty acid and glucose oxidation, lipid accumulation, impaired ATP production	MRI and metabolomic studies show increased myocardial lipid content and altered acylcarnitine profiles [28,33]
Mitochondrial Structural Abnormalities	Swollen mitochondria, cristae disruption, impaired fission/fusion balance (↑ DRP1, ↓ MFN2/OPA1)	Electron microscopy of human HFpEF myocardium; proteomic evidence of reduced ETC complex activity [36,39]
Mitochondrial Dysfunction and Energy Deficit	↓ Oxidative phosphorylation, ↓ Complex I/IV activity, ↑ ROS	Downregulation of TCA, ETC enzymes; decreased Ca^2+^ buffering and mitochondrial respiration [41]
Inflammation and Oxidative Stress	↑ IL-6, TNF-α, CRP, macrophage infiltration; endothelial dysfunction via NOX2 and eNOS uncoupling	Increased systemic and myocardial inflammatory markers [49,53]
Endothelial and Skeletal Muscle Mitochondrial Dysfunction	Reduced NO bioavailability, impaired vasodilation, ↓ skeletal muscle oxidative capacity	Reduced MFN2 and mitochondrial content in skeletal muscle of HFpEF patients [46]

↓ decrease, ↑ increase.

## Data Availability

No new data were created or analyzed in this study. Data sharing is not applicable to this article.

## References

[1] Savarese G., Becher P.M., Lund L.H., Seferovic P., Rosano G.M.C., Coats A.J.S. (2023). Global burden of heart failure: A comprehensive and updated review of epidemiology. Cardiovasc. Res..

[2] Berg D.D., Samsky M.D., Velazquez E.J., Duffy C.I., Gurmu Y., Braunwald E., Morrow D.A., DeVore A.D. (2021). Efficacy and Safety of Sacubitril/Valsartan in High-Risk Patients in the PIONEER-HF Trial. Circ. Heart Fail..

[3] Xanthopoulos A., Triposkiadis F., Starling R.C. (2018). Heart failure with preserved ejection fraction: Classification based upon phenotype is essential for diagnosis and treatment. Trends Cardiovasc. Med..

[4] Stoicescu L., Crisan D., Morgovan C., Avram L., Ghibu S. (2024). Heart Failure with Preserved Ejection Fraction: The Pathophysiological Mechanisms behind the Clinical Phenotypes and the Therapeutic Approach. Int. J. Mol. Sci..

[5] Omote K., Verbrugge F.H., Borlaug B.A. (2022). Heart Failure with Preserved Ejection Fraction: Mechanisms and Treatment Strategies. Annu. Rev. Med..

[6] Dunlay S.M., Roger V.L., Redfield M.M. (2017). Epidemiology of heart failure with preserved ejection fraction. Nat. Rev. Cardiol..

[7] Paulus W.J., Tschope C., Sanderson J.E., Rusconi C., Flachskampf F.A., Rademakers F.E., Marino P., Smiseth O.A., De Keulenaer G., Leite-Moreira A.F. (2007). How to diagnose diastolic heart failure: A consensus statement on the diagnosis of heart failure with normal left ventricular ejection fraction by the Heart Failure and Echocardiography Associations of the European Society of Cardiology. Eur. Heart J..

[8] Borlaug B.A. (2020). Evaluation and management of heart failure with preserved ejection fraction. Nat. Rev. Cardiol..

[9] Lam C.S., Donal E., Kraigher-Krainer E., Vasan R.S. (2011). Epidemiology and clinical course of heart failure with preserved ejection fraction. Eur. J. Heart Fail..

[10] Paulus W.J., Tschope C. (2013). A novel paradigm for heart failure with preserved ejection fraction: Comorbidities drive myocardial dysfunction and remodeling through coronary microvascular endothelial inflammation. J. Am. Coll. Cardiol..

[11] Franssen C., Chen S., Hamdani N., Paulus W.J. (2016). From comorbidities to heart failure with preserved ejection fraction: A story of oxidative stress. Heart.

[12] Weerts J., Mourmans S.G.J., Barandiaran Aizpurua A., Schroen B.L.M., Knackstedt C., Eringa E., Houben A., van Empel V.P.M. (2022). The Role of Systemic Microvascular Dysfunction in Heart Failure with Preserved Ejection Fraction. Biomolecules.

[13] Peoples J.N., Saraf A., Ghazal N., Pham T.T., Kwong J.Q. (2019). Mitochondrial dysfunction and oxidative stress in heart disease. Exp. Mol. Med..

[14] Velollari O., Rommel K.P., Kresoja K.P., Lurz P., Gori T. (2025). Focusing on microvascular function in heart failure with preserved ejection fraction. Heart Fail. Rev..

[15] Bhattarai N., Scott I. (2024). In the heart and beyond: Mitochondrial dysfunction in heart failure with preserved ejection fraction (HFpEF). Curr. Opin. Pharmacol..

[16] Del Campo A., Perez G., Castro P.F., Parra V., Verdejo H.E. (2021). Mitochondrial function, dynamics and quality control in the pathophysiology of HFpEF. Biochim. Biophys. Acta Mol. Basis Dis..

[17] Kumar A.A., Kelly D.P., Chirinos J.A. (2019). Mitochondrial Dysfunction in Heart Failure With Preserved Ejection Fraction. Circulation.

[18] Daou D., Gillette T.G., Hill J.A. (2023). Inflammatory Mechanisms in Heart Failure with Preserved Ejection Fraction. Physiology.

[19] Werbner B., Tavakoli-Rouzbehani O.M., Fatahian A.N., Boudina S. (2023). The dynamic interplay between cardiac mitochondrial health and myocardial structural remodeling in metabolic heart disease, aging, and heart failure. J. Cardiovasc. Aging.

[20] Shires S.E., Gustafsson A.B. (2015). Mitophagy and heart failure. J. Mol. Med..

[21] Kirkman D.L., Robinson A.T., Rossman M.J., Seals D.R., Edwards D.G. (2021). Mitochondrial contributions to vascular endothelial dysfunction, arterial stiffness, and cardiovascular diseases. Am. J. Physiol. Heart Circ. Physiol..

[22] Liu M., Lv J., Pan Z., Wang D., Zhao L., Guo X. (2022). Mitochondrial dysfunction in heart failure and its therapeutic implications. Front. Cardiovasc. Med..

[23] Lopaschuk G.D., Karwi Q.G., Tian R., Wende A.R., Abel E.D. (2021). Cardiac Energy Metabolism in Heart Failure. Circ. Res..

[24] Karwi Q.G., Uddin G.M., Ho K.L., Lopaschuk G.D. (2018). Loss of Metabolic Flexibility in the Failing Heart. Front. Cardiovasc. Med..

[25] Doenst T., Nguyen T.D., Abel E.D. (2013). Cardiac metabolism in heart failure: Implications beyond ATP production. Circ. Res..

[26] Hiebert J.B., Shen Q., Thimmesch A., Pierce J. (2016). Impaired Myocardial Bioenergetics in HFpEF and the Role of Antioxidants. Open Cardiovasc. Med. J..

[27] Paraskevaidis I., Tsougos E., Kourek C. (2025). One Syndrome, Many Faces: A Unified Perspective on Heart Failure Phenotypes. Int. J. Mol. Sci..

[28] Wu C.K., Lee J.K., Hsu J.C., Su M.M., Wu Y.F., Lin T.T., Lan C.W., Hwang J.J., Lin L.Y. (2020). Myocardial adipose deposition and the development of heart failure with preserved ejection fraction. Eur. J. Heart Fail..

[29] Chiu H.C., Kovacs A., Ford D.A., Hsu F.F., Garcia R., Herrero P., Saffitz J.E., Schaffer J.E. (2001). A novel mouse model of lipotoxic cardiomyopathy. J. Clin. Investig..

[30] Shehadeh L.A., Robleto E., Lopaschuk G.D. (2025). Cardiac energy substrate utilization in heart failure with preserved ejection fraction: Reconciling conflicting evidence on fatty acid and glucose metabolism. Am. J. Physiol. Heart Circ. Physiol..

[31] Sun Q., Guven B., Wagg C.S., Almeida de Oliveira A., Silver H., Zhang L., Chen B., Wei K., Ketema E.B., Karwi Q.G. (2024). Mitochondrial fatty acid oxidation is the major source of cardiac adenosine triphosphate production in heart failure with preserved ejection fraction. Cardiovasc. Res..

[32] Tong D., Schiattarella G.G., Jiang N., Altamirano F., Szweda P.A., Elnwasany A., Lee D.I., Yoo H., Kass D.A., Szweda L.I. (2021). NAD^+^ Repletion Reverses Heart Failure With Preserved Ejection Fraction. Circ. Res..

[33] Yoshihisa A., Watanabe S., Yokokawa T., Misaka T., Sato T., Suzuki S., Oikawa M., Kobayashi A., Takeishi Y. (2017). Associations between acylcarnitine to free carnitine ratio and adverse prognosis in heart failure patients with reduced or preserved ejection fraction. ESC Heart Fail..

[34] Mori J., Basu R., McLean B.A., Das S.K., Zhang L., Patel V.B., Wagg C.S., Kassiri Z., Lopaschuk G.D., Oudit G.Y. (2012). Agonist-induced hypertrophy and diastolic dysfunction are associated with selective reduction in glucose oxidation: A metabolic contribution to heart failure with normal ejection fraction. Circ. Heart Fail..

[35] Ye L., Fu X., Li Q. (2025). Mitochondrial Quality Control in Health and Disease. MedComm.

[36] Meddeb M., Koleini N., Binek A., Keykhaei M., Darehgazani R., Kwon S., Aboaf C., Margulies K.B., Bedi K.C., Lehar M. (2024). Myocardial ultrastructure of human heart failure with preserved ejection fraction. Nat. Cardiovasc. Res..

[37] Uchikado Y., Ikeda Y., Ohishi M. (2022). Current Understanding of the Pivotal Role of Mitochondrial Dynamics in Cardiovascular Diseases and Senescence. Front. Cardiovasc. Med..

[38] Givvimani S., Munjal C., Tyagi N., Sen U., Metreveli N., Tyagi S.C. (2012). Mitochondrial division/mitophagy inhibitor (Mdivi) ameliorates pressure overload induced heart failure. PLoS ONE.

[39] Jani V.P., Yoo E.J., Binek A., Guo A., Kim J.S., Aguilan J., Keykhaei M., Jenkin S.R., Sidoli S., Sharma K. (2025). Myocardial Proteome in Human Heart Failure With Preserved Ejection Fraction. J. Am. Heart Assoc..

[40] Santulli G., Xie W., Reiken S.R., Marks A.R. (2015). Mitochondrial calcium overload is a key determinant in heart failure. Proc. Natl. Acad. Sci. USA.

[41] Cui X., Spanos M., Zhao C., Wan W., Cui C., Wang L., Xiao J. (2025). Mitochondrial Dysfunction in HFpEF: Potential Interventions Through Exercise. J. Cardiovasc. Transl. Res..

[42] Chaanine A.H., Joyce L.D., Stulak J.M., Maltais S., Joyce D.L., Dearani J.A., Klaus K., Nair K.S., Hajjar R.J., Redfield M.M. (2019). Mitochondrial Morphology, Dynamics, and Function in Human Pressure Overload or Ischemic Heart Disease With Preserved or Reduced Ejection Fraction. Circ. Heart Fail..

[43] Landes T., Emorine L.J., Courilleau D., Rojo M., Belenguer P., Arnaune-Pelloquin L. (2010). The BH3-only Bnip3 binds to the dynamin Opa1 to promote mitochondrial fragmentation and apoptosis by distinct mechanisms. EMBO Rep..

[44] Bode D., Wen Y., Hegemann N., Primessnig U., Parwani A., Boldt L.H., MPieske B., RHeinzel F., Hohendanner F. (2020). Oxidative Stress and Inflammatory Modulation of Ca^2+^ Handling in Metabolic HFpEF-Related Left Atrial Cardiomyopathy. Antioxidants.

[45] Zhang W., Zhang H., Yao W., Li L., Niu P., Huo Y., Tan W. (2020). Morphometric, Hemodynamic, and Multi-Omics Analyses in Heart Failure Rats with Preserved Ejection Fraction. Int. J. Mol. Sci..

[46] Molina A.J., Bharadwaj M.S., Van Horn C., Nicklas B.J., Lyles M.F., Eggebeen J., Haykowsky M.J., Brubaker P.H., Kitzman D.W. (2016). Skeletal Muscle Mitochondrial Content, Oxidative Capacity, and Mfn2 Expression Are Reduced in Older Patients With Heart Failure and Preserved Ejection Fraction and Are Related to Exercise Intolerance. JACC Heart Fail..

[47] Martinez C.S., Zheng A., Xiao Q. (2024). Mitochondrial Reactive Oxygen Species Dysregulation in Heart Failure with Preserved Ejection Fraction: A Fraction of the Whole. Antioxidants.

[48] Sorop O., Heinonen I., van Kranenburg M., van de Wouw J., de Beer V.J., Nguyen I.T.N., Octavia Y., van Duin R.W.B., Stam K., van Geuns R.J. (2018). Multiple common comorbidities produce left ventricular diastolic dysfunction associated with coronary microvascular dysfunction, oxidative stress, and myocardial stiffening. Cardiovasc. Res..

[49] Curran F.M., Bhalraam U., Mohan M., Singh J.S., Anker S.D., Dickstein K., Doney A.S., Filippatos G., George J., Metra M. (2021). Neutrophil-to-lymphocyte ratio and outcomes in patients with new-onset or worsening heart failure with reduced and preserved ejection fraction. ESC Heart Fail..

[50] Burlew B.S., Weber K.T. (2002). Cardiac fibrosis as a cause of diastolic dysfunction. Herz.

[51] Loster H., Grunder W., Keller T., Seim H., Grommisch J., Muller F. (1990). The effect of L-carnitine on the energy metabolism of isolated rat heart perfused by Langendorff’s method using 31P-NMR spectroscopy. Z. Med. Lab. Diagn..

[52] Zhazykbayeva S., Pabel S., Mugge A., Sossalla S., Hamdani N. (2020). The molecular mechanisms associated with the physiological responses to inflammation and oxidative stress in cardiovascular diseases. Biophys. Rev..

[53] Hulsmans M., Sager H.B., Roh J.D., Valero-Munoz M., Houstis N.E., Iwamoto Y., Sun Y., Wilson R.M., Wojtkiewicz G., Tricot B. (2018). Cardiac macrophages promote diastolic dysfunction. J. Exp. Med..

[54] Griendling K.K., Sorescu D., Ushio-Fukai M. (2000). NAD(P)H oxidase: Role in cardiovascular biology and disease. Circ. Res..

[55] Gevaert A.B., Boen J.R.A., Segers V.F., Van Craenenbroeck E.M. (2019). Heart Failure With Preserved Ejection Fraction: A Review of Cardiac and Noncardiac Pathophysiology. Front. Physiol..

[56] Preiser J.C. (2012). Oxidative stress. J. Parenter. Enteral Nutr..

[57] Mongirdiene A., Skrodenis L., Varoneckaite L., Mierkyte G., Gerulis J. (2022). Reactive Oxygen Species Induced Pathways in Heart Failure Pathogenesis and Potential Therapeutic Strategies. Biomedicines.

[58] Perez-Gomez R., Magnin V., Mihajlovic Z., Slaninova V., Krejci A. (2020). Downregulation of respiratory complex I mediates major signalling changes triggered by TOR activation. Sci. Rep..

[59] Pinzon-Fernandez M.V., Saavedra-Torres J.S., Lopez Garzon N.A., Pachon-Bueno J.S., Tamayo-Giraldo F.J., Rojas Gomez M.C., Arias-Intriago M., Gaibor-Pazmino A., Lopez-Cortes A., Izquierdo-Condoy J.S. (2025). NLRP3 and beyond: Inflammasomes as central cellular hub and emerging therapeutic target in inflammation and disease. Front. Immunol..

[60] Pandey A., Parashar A., Kumbhani D., Agarwal S., Garg J., Kitzman D., Levine B., Drazner M., Berry J. (2015). Exercise training in patients with heart failure and preserved ejection fraction: Meta-analysis of randomized control trials. Circ. Heart Fail..

[61] Kitzman D.W., Brubaker P., Morgan T., Haykowsky M., Hundley G., Kraus W.E., Eggebeen J., Nicklas B.J. (2016). Effect of Caloric Restriction or Aerobic Exercise Training on Peak Oxygen Consumption and Quality of Life in Obese Older Patients With Heart Failure With Preserved Ejection Fraction: A Randomized Clinical Trial. JAMA.

[62] Hummel S.L., Seymour E.M., Brook R.D., Sheth S.S., Ghosh E., Zhu S., Weder A.B., Kovacs S.J., Kolias T.J. (2013). Low-sodium DASH diet improves diastolic function and ventricular-arterial coupling in hypertensive heart failure with preserved ejection fraction. Circ. Heart Fail..

[63] Kjeldsen S.E., von Lueder T.G., Smiseth O.A., Wachtell K., Mistry N., Westheim A.S., Hopper I., Julius S., Pitt B., Reid C.M. (2020). Medical Therapies for Heart Failure With Preserved Ejection Fraction. Hypertension.

[64] McMurray J.J., Packer M., Desai A.S., Gong J., Lefkowitz M.P., Rizkala A.R., Rouleau J.L., Shi V.C., Solomon S.D., Swedberg K. (2014). Angiotensin-neprilysin inhibition versus enalapril in heart failure. N. Engl. J. Med..

[65] Solomon S.D., McMurray J.J.V., Claggett B., de Boer R.A., DeMets D., Hernandez A.F., Inzucchi S.E., Kosiborod M.N., Lam C.S.P., Martinez F. (2022). Dapagliflozin in Heart Failure with Mildly Reduced or Preserved Ejection Fraction. N. Engl. J. Med..

[66] Anker S.D., Butler J., Filippatos G., Ferreira J.P., Bocchi E., Bohm M., Brunner-La Rocca H.P., Choi D.J., Chopra V., Chuquiure-Valenzuela E. (2021). Empagliflozin in Heart Failure with a Preserved Ejection Fraction. N. Engl. J. Med..

[67] Reddy B.M., Weintraub H.S., Schwartzbard A.Z. (2010). Ranolazine: A new approach to treating an old problem. Tex. Heart Inst. J..

[68] Campos J.C., Queliconi B.B., Bozi L.H.M., Bechara L.R.G., Dourado P.M.M., Andres A.M., Jannig P.R., Gomes K.M.S., Zambelli V.O., Rocha-Resende C. (2017). Exercise reestablishes autophagic flux and mitochondrial quality control in heart failure. Autophagy.

[69] Bo H., Jiang N., Ma G., Qu J., Zhang G., Cao D., Wen L., Liu S., Ji L.L., Zhang Y. (2008). Regulation of mitochondrial uncoupling respiration during exercise in rat heart: Role of reactive oxygen species (ROS) and uncoupling protein 2. Free Radic. Biol. Med..

[70] Xia J., Nong Y., Teng J., Mohammed S.A., Liu J., Pang Y., Costantino S., Ruschitzka F., Hamdani N., Abdellatif M. (2025). Unlocking metabolic flexibility in heart failure with preserved ejection fraction: Bridging fundamental mechanisms to clinical innovation. iScience.

[71] Deng Y., Xie M., Li Q., Xu X., Ou W., Zhang Y., Xiao H., Yu H., Zheng Y., Liang Y. (2021). Targeting Mitochondria-Inflammation Circuit by beta-Hydroxybutyrate Mitigates HFpEF. Circ. Res..

[72] Gopalasingam N., Berg-Hansen K., Christensen K.H., Ladefoged B.T., Poulsen S.H., Andersen M.J., Borlaug B.A., Nielsen R., Moller N., Wiggers H. (2024). Randomized Crossover Trial of 2-Week Ketone Ester Treatment in Patients With Type 2 Diabetes and Heart Failure With Preserved Ejection Fraction. Circulation.

[73] Cinti F., Laborante R., Cappannoli L., Morciano C., Gugliandolo S., Pontecorvi A., Burzotta F., Donniacuo M., Cappetta D., Patti G. (2025). The effects of SGLT2i on cardiac metabolism in patients with HFpEF: Fact or fiction?. Cardiovasc. Diabetol..

[74] Kolijn D., Pabel S., Tian Y., Lodi M., Herwig M., Carrizzo A., Zhazykbayeva S., Kovacs A., Fulop G.A., Falcao-Pires I. (2021). Empagliflozin improves endothelial and cardiomyocyte function in human heart failure with preserved ejection fraction via reduced pro-inflammatory-oxidative pathways and protein kinase Galpha oxidation. Cardiovasc. Res..

[75] Giaccari A. (2019). Sodium-glucose co-transporter inhibitors: Medications that mimic fasting for cardiovascular prevention. Diabetes Obes. Metab..

[76] Hu X., Li D., Chen W., Kuang H., Yang D., Gong Z., Long Y., Liu G., Wang K., Xia M. (2025). Sodium Glucose Transporter 2 Inhibitor Protects Against Heart Failure With Preserved Ejection Fraction: Preclinical “2-Hit” Model Reveals Autophagy Enhancement Via AMP-Activated Protein Kinase/Mammalian Target of Rapamycin Complex 1 Pathway. J. Am. Heart Assoc..

[77] Bode D., Semmler L., Wakula P., Hegemann N., Primessnig U., Beindorff N., Powell D., Dahmen R., Ruetten H., Oeing C. (2021). Dual SGLT-1 and SGLT-2 inhibition improves left atrial dysfunction in HFpEF. Cardiovasc. Diabetol..

[78] da Silva T.G., Ribeiro R.S., Mencalha A.L., de Souza Fonseca A. (2023). Photobiomodulation at molecular, cellular, and systemic levels. Lasers Med. Sci..

[79] Hamblin M.R. (2017). Mechanisms and applications of the anti-inflammatory effects of photobiomodulation. AIMS Biophys..

[80] Dashdorj A., Jyothi K.R., Lim S., Jo A., Nguyen M.N., Ha J., Yoon K.S., Kim H.J., Park J.H., Murphy M.P. (2013). Mitochondria-targeted antioxidant MitoQ ameliorates experimental mouse colitis by suppressing NLRP3 inflammasome-mediated inflammatory cytokines. BMC Med..

[81] Goh K.Y., He L., Song J., Jinno M., Rogers A.J., Sethu P., Halade G.V., Rajasekaran N.S., Liu X., Prabhu S.D. (2019). Mitoquinone ameliorates pressure overload-induced cardiac fibrosis and left ventricular dysfunction in mice. Redox Biol..

[82] Rossman M.J., Santos-Parker J.R., Steward C.A.C., Bispham N.Z., Cuevas L.M., Rosenberg H.L., Woodward K.A., Chonchol M., Gioscia-Ryan R.A., Murphy M.P. (2018). Chronic Supplementation With a Mitochondrial Antioxidant (MitoQ) Improves Vascular Function in Healthy Older Adults. Hypertension.

[83] Chakraborty A., Minor K.E., Nizami H.L., Chiao Y.A., Lee C.F. (2022). Harnessing NAD^+^ Metabolism as Therapy for Cardiometabolic Diseases. Curr. Heart Fail. Rep..

